# Galactosylated GAG from a Snail Leads to Better Clot Buster

**DOI:** 10.1021/acscentsci.6c00639

**Published:** 2026-05-12

**Authors:** Ramiz Demir, Lara K. Mahal

**Affiliations:** Department of Chemistry, 3158 University of Alberta, Edmonton, Alberta T6G 2G2, Canada

## Abstract

A novel galactosylated glycosaminoglycan, identified by Wu and colleagues, brings new opportunities to target the coagulation cascade without enhanced bleeding.

## Graphical abstract

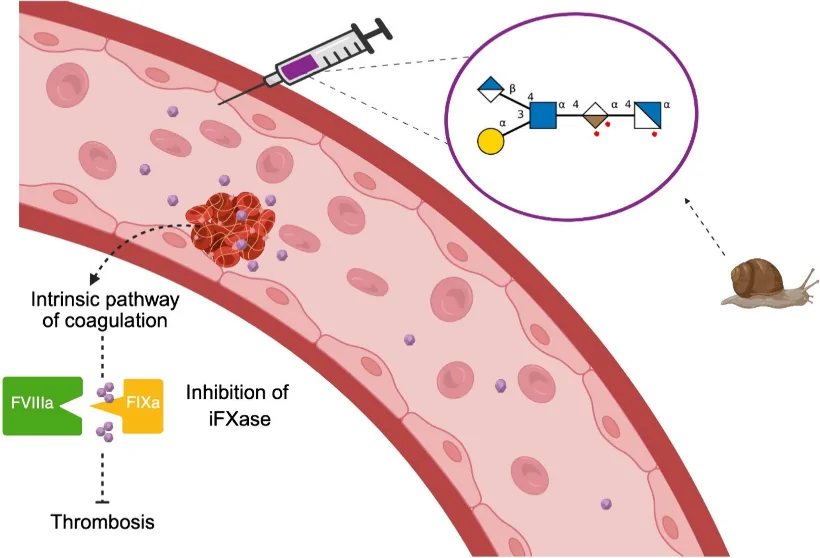

For almost a century, the glycosaminoglycan (GAG) heparin has been used as an anticoagulant, despite the increased bleeding risk it causes. In a recent issue of *ACS Central Science*, Wu and colleagues isolate and characterize a new galactosylated GAG from a snail that targets internal clotting without increased bleeding risk, providing a new basis for anticoagulant development.

In a recent issue of *ACS Central Science*, Wu and colleagues isolate and characterize a new galactosylated GAG from a snail that targets internal clotting without increased bleeding risk, providing a new basis for anticoagulant development.

Thrombosis is a serious life-threatening condition in which blood clots within blood vessels cause damage.^1,2^ A well-known example is deep vein thrombosis (DVT), which can occur due to sitting too long on an airplane or other sedentary behaviors. If these clots release from the vein, they can cause a pulmonary embolism, which can be fatal. Standard treatment for clots includes the use of anticoagulants such as heparin or warfarin; however, these drugs come with significant concerns due to increased bleeding.^3^ In a recent issue of *ACS Central Science*, Mingyi Wu and co-workers identified a GAG with a unique galactosylated structure from the land snail *Camaena cicatricosa* (CCG) that enables anticoagulant activity without enhanced bleeding in animal models (Figure 1). This natural product has both major and minor GAG polymers with differences in sulfation patterns. While the exact structural requirements leading to anticoagulant activity remain to be elucidated, this paper points the way toward the creation of more precise GAG-based anticoagulants.

**Figure 1:**
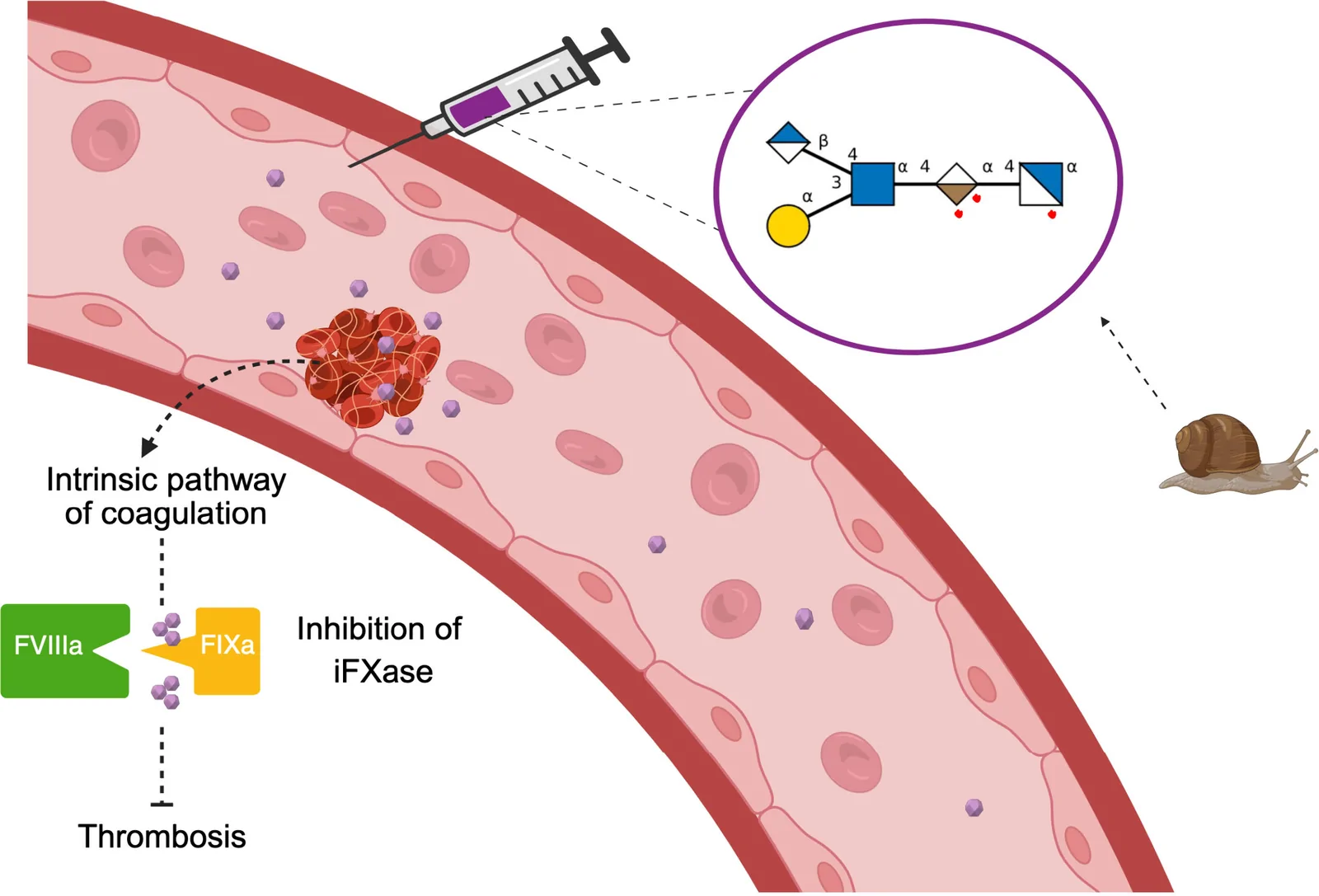
Galactosylated GAGs from snail inhibit the iFXase complex, preventing thrombosis. A fragment of the snail GAG is shown in the purple circle using the Symbolic Nomenclature for Glycans (yellow circle: galactose; blue square: *N-*acetylglucosamine; blue/white diamond: glucuronic acid; brown/white diamond: iduronic acid; blue/white square: glucosamine; red dots: sulfation).

One of the most well-known anticoagulants in clinical use is heparin, a heavily sulfated GAG that was identified ∼100 years ago. A natural product, it is commonly isolated from pigs and works by inhibiting thrombin (FIIa) and/or FXa within the coagulation cascade.^4^ Several issues exist with the use of heparin clinically. First, contamination of heparin by other GAGs can lead to serious side effects as occurred in the early 2000s. Second, heparin use causes excessive bleeding. Over the past 20 years, there have been increased efforts to create heparin mimetics and biosynthetic versions of this GAG to combat both issues.^5^ The drug Arixtra® is a synthetic pentasaccharide based on heparin that is used to treat deep vein thrombosis. However, it also can cause bleeding as a side effect.

Improvements in understanding the coagulation cascade have provided new targets for anticoagulants that might not induce bleeding.^6^ The discovery that the “intrinsic” pathway, which involves conversion of Factor XIa to FIXa, which then binds to FVIIIa, creating the iFXase complex, may regulate thrombosis without impacting hemostasis (i.e., the ability of blood to clot in a wound) has resulted in a series of therapeutics targeting this part of the pathway. Several of these are in clinical trials, although none are yet in clinical use. In contrast, heparin and most currently used anticoagulants target the coagulation cascade downstream of where the intrinsic and extrinsic (i.e., trauma-based) parts of the pathway converge and thus impact bleeding. By leveraging natural product isolation and structural determination, this new work by Lin et al. finds novel GAG motifs that target the iFXase complex, opening up new possibilities for anticoagulant therapies.

By leveraging natural product isolation and structural determination, this new work by Lin et al. finds novel GAG motifs that target the iFXase complex, opening up new possibilities for anticoagulant therapies.

While from a practical standpoint, isolation of snail GAGs may be problematic, these snail GAGs can act as a new medicinal chemistry lead. In the future, structure–function studies and advances in synthetic chemistry may lead to a new generation of GAG-based anticoagulants.
